# Determinants of gas exchange during extracorporeal CO_2_ removal using a novel pump-driven venovenous gas exchange system in a minimally invasive setting

**DOI:** 10.1186/cc14354

**Published:** 2015-03-16

**Authors:** A Hermann, K Riss, P Schellongowski, A Bojic, P Wohlfarth, O Robak, W Sperr, T Staudinger

**Affiliations:** 1 Medical University of Vienna, Austria

## Introduction

Pump-driven venovenous extracorporeal CO_2_ removal (ECCO_2_-R) increasingly takes root in hypercapnic lung failure to minimize ventilation invasiveness or to avoid intubation. A recently developed miniaturized device consisting of a centrifugal pump and a membrane ventilator (iLA Activve®; Novalung, Germany) allows effective decarboxylation via a jugular double lumen cannula. So far no data on gas exchange in this setting exist to date.

## Methods

We included 10 patients receiving iLA Activve® due to hypercapnic respiratory failure as bridge-to-transplant or obstructive lung disease. Sweep gas flow was increased in steps from 1 to 14 l/ minute at constant blood flow (phase 1). Similarly, blood flow was gradually increased at constant sweep gas flow (phase 2). At each step, gas transfer via the membrane as well as arterial blood gas samples were obtained.

## Results

**Figure 1:**
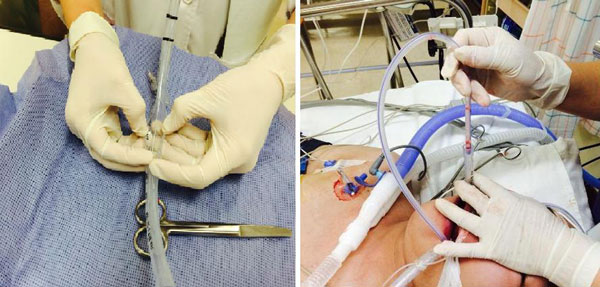
PaO_2_ with increasing blood flow (*P <0.0001).

During phase 1, we observed a significant increase in CO_2 _transfer together with a decrease in PaCO_2_ levels from a median of 66 mmHg (range 46 to 85) to 49 (31 to 65) mmHg from 1 to 14 l/ minute sweep gas flow, while arterial oxygenation deteriorated with high sweep gas flow rates. During phase 2, oxygen transfer significantly increased leading to an increase in PaO_2_ from 67 (49 to 87) at 0.5 l/ minute to 117 (66 to 305) mmHg at 2.0 l/minute. Higher blood flow rates also significantly enhanced decarboxylation. Increasing blood flow to 2.0 l/minute led to negative suction pressures of more than -100 mmHg and signs of hemolysis. See Figure 1.

## Conclusion

Increasing sweep gas flow results in effective CO_2_ removal which can be further reinforced by raising blood flow. The clinically relevant oxygenation effect even in this setting of low invasivity could broaden the range of indications towards hypercapnic lung failure with mild to moderate hypoxia.

